# The Stethoscope, and Virginia Medical Gazette, a Monthly Journal of Medicine and the Collateral Sciences

**Published:** 1851-04

**Authors:** 


THE STETHOSCOPE, *and Virginia Medical Gazette*, a monthly Journal of Medicine and the Collateral sciences. Edited by P. Claiborne Gooch, M. D.

We owe our able confrère of Richmond, Va., an explanation of our silence, heretofore, respecting his new periodical, and he will pardon our apparent remissness, when we inform him that owing to a mistake at the post-office, we did not receive any of his numbers in time to refer to them earlier than now.

We give the Stethoscope a cordial welcome, and wish it a prosperous career.

The Stethoscope is published at Richmond, Va., at three dollars per annum. B.

---

**OMISSIONS—Several valuable papers have been crowded out of this number.** **B.**

